# The signature of liver cancer in immune cells DNA methylation

**DOI:** 10.1186/s13148-017-0436-1

**Published:** 2018-01-18

**Authors:** Yonghong Zhang, Sophie Petropoulos, Jinhua Liu, David Cheishvili, Rudy Zhou, Sergiy Dymov, Kang Li, Ning Li, Moshe Szyf

**Affiliations:** 1 Beijing Youan Hospital, Capital Medical School, Beijing, China; 2 Department of Pharmacology and Therapeutics, McGill University, 3655 Sir William Osler Promenade, Montreal, Quebec H3G 1Y6 Canada; 3 Deparment of Clinical Science, Karolinska Institutet, Alfred Nobels Allé 8, 141 52 Huddinge, Sweden; 4 Montreal EpiTerapia Inc., 4567 Cecile, H9K1N2, Montreal, QC Canada

**Keywords:** DNA methylation, Hepatocellular carcinoma, Peripheral white blood cells, Immune functions

## Abstract

**Background:**

The idea that changes to the host immune system are critical for cancer progression was proposed a century ago and recently regained experimental support.

**Results:**

Herein, the hypothesis that hepatocellular carcinoma (HCC) leaves a molecular signature in the host peripheral immune system was tested by profiling DNA methylation in peripheral blood mononuclear cells (PBMC) and T cells from a discovery cohort (*n* = 69) of healthy controls, chronic hepatitis, and HCC using Illumina 450K platform and was validated in two validation sets (*n* = 80 and *n* = 48) using pyrosequencing.

**Conclusions:**

The study reveals a broad signature of hepatocellular carcinoma in PBMC and T cells DNA methylation which discriminates early HCC stage from chronic hepatitis B and C and healthy controls, intensifies with progression of HCC, and is highly enriched in immune function-related genes such as *PD-1*, a current cancer immunotherapy target. These data also support the feasibility of using these profiles for early detection of HCC.

**Electronic supplementary material:**

The online version of this article (10.1186/s13148-017-0436-1) contains supplementary material, which is available to authorized users.

## Backgrounds

The idea that host immuno-surveillance plays an important role in tumorigenesis by eliminating tumor cells and suppressing tumor growth has been proposed by Paul Ehrlich [1, 2] more than a century ago and has fallen out of favor. However, accumulating data from both animal and human clinical studies suggest that the host immune system plays an important role in tumorigenesis through “immuno-editing” which involves three stages: elimination, equilibrium, and escape [3–5]. Presence of tumor infiltrating cytotoxic CD8+ T cells was associated with better prognosis in several clinical studies of human regressive melanoma [6–11], esophageal [12], ovarian [13, 14], and colorectal cancer [15–17]. The immune system is believed to be responsible for the phenomenon of cancer dormancy when circulating cancer cells are detectable in the absence of clinical symptoms [18, 19].

DNA methylation, a covalent modification of DNA, which is a primary mechanism of epigenetic regulation of genome function, is ubiquitously altered in tumors [18, 20–22] including hepatocellular carcinoma (HCC) [23]. Molecular analysis of cancer including DNA methylation is mainly focused on tumors and biomaterial originating in tumor including tumor DNA in plasma [24, 25], circulating tumor cells [26], and the tumor-host microenvironment [27, 28]. The prevailing and widely accepted hypothesis is that molecular changes that drive cancer initiation and progression originate primarily in the tumor itself and that relevant changes in the host occur primarily in the tumor microenvironment [27, 29]. The identity of immune cells in the tumor microenvironment has attracted, therefore, significant attention [30, 31]. Interestingly, recent DNA methylation and transcriptome analysis of tumors revealed tumor stage-specific immune signatures of infiltrating lymphocytes [29, 32]. However, these signatures represent targeted immune cells in the tumor microenvironment, and utilization of such signatures for early diagnosis requires invasive procedures. The tumor-infiltrating immune cells represent only a minor fraction of peripheral blood cells [33–36].

Global DNA methylation changes were previously reported in leukocytes, and EWAS (epigenome-wide association studies) studies revealed differences in DNA methylation in leukocytes from bladder, head and neck, and ovarian cancer, and these differences were independent of differences in white blood cell distribution [37]. Differential methylation of 53 CG sites that did not show evidence of association with blood cell composition was found to associate with ovarian cancer risk in blood DNA [38]. A recent study demonstrated association between the state of methylation of multiple CG site in six genes and colorectal cancer in peripheral blood leukocytes as well as an interaction with diet [39]. An EWAS on 48 matched case-controlled pairs in a nested case-control study within a 22-year follow-up cohort of hepatitis B (HepB) carriers revealed methylation variable positions that were associated with progression to HCC and were predictive of the risk of early-onset HCC well before appearance of clinical symptoms [40]. These data provide support for the hypothesis that cancer-specific DNA methylation differences exist in white blood cells; however, it is possible that these just reflect stochastic alterations in DNA methylation in somatic tissues that are associated with cancer.

The question of whether the peripheral host immune system exhibits a distinct DNA methylation response to the cancer state that intensifies in advanced stages of cancer has not been addressed. Addressing this question is essential for understanding the potential importance of epigenetic reprogramming of the immune system in cancer. Does epigenetic reprogramming occur in the immune system during cancer progression and does it play a causal role in HCC progression? If indeed progression of cancer involves epigenetic reprogramming of the host immune system, this has important implications for both therapeutics and diagnostics.

HCC is the fifth most common cancer worldwide [41]. It is particularly prevalent in Asia, and its occurrence is highest in areas where hepatitis B is prevalent, indicating a possible causal relationship [42]. Here, we hypothesize that HCC progression is associated with distinct DNA methylation profiles in the host peripheral immune cells. Since HCC originates in patients with an ongoing inflammatory chronic viral infection, the critical challenge is to delineate DNA methylation markers that differentiate between cancer and the underlying chronic inflammatory liver disease. We hypothesize here that HCC exhibits a DNA methylation profile that is distinct from chronic hepatitis and that there is a clear boundary in the evolution of the DNA methylation profile of white blood cells between the earliest stages of HCC and chronic hepatitis

Our study demonstrates for the first time broad DNA methylation profiles for HCC in peripheral blood mononuclear cells (PBMC) and T cells that are different from controls as well as hepatitis B and C; the differences are intensified during cancer progression. There is a significant overlap between DNA methylation profiles delineated in white blood cells and T cells. Four genes that were differentially methylated in T cells from HCC patients in the discovery cohort were validated by pyrosequencing of T cells DNA in a separate cohort of patients (*n* = 79) and one gene *STAP1* was validated in a third cohort (*n* = 48). HCC DNA methylation profiles are highly enriched in immune functions including genes such as *Programmed cell Death 1* (*PD-1*), a negative regulator of T cell immune response that is an important target in current cancer immunotherapy [43] and show no significant overlap with the DNA methylation profiles of previously described HCC tumors [23]. These data provide proof of principle that there are molecular changes in the host immune cells DNA in HCC. This has important implications for our understanding of the mechanisms of the disease and its treatment as well as for noninvasive diagnostics of cancer in white blood cells DNA.

## Results

### Correlation between quantitative distribution of site-specific DNA methylation levels and progression of HCC

**Fig. 1:**
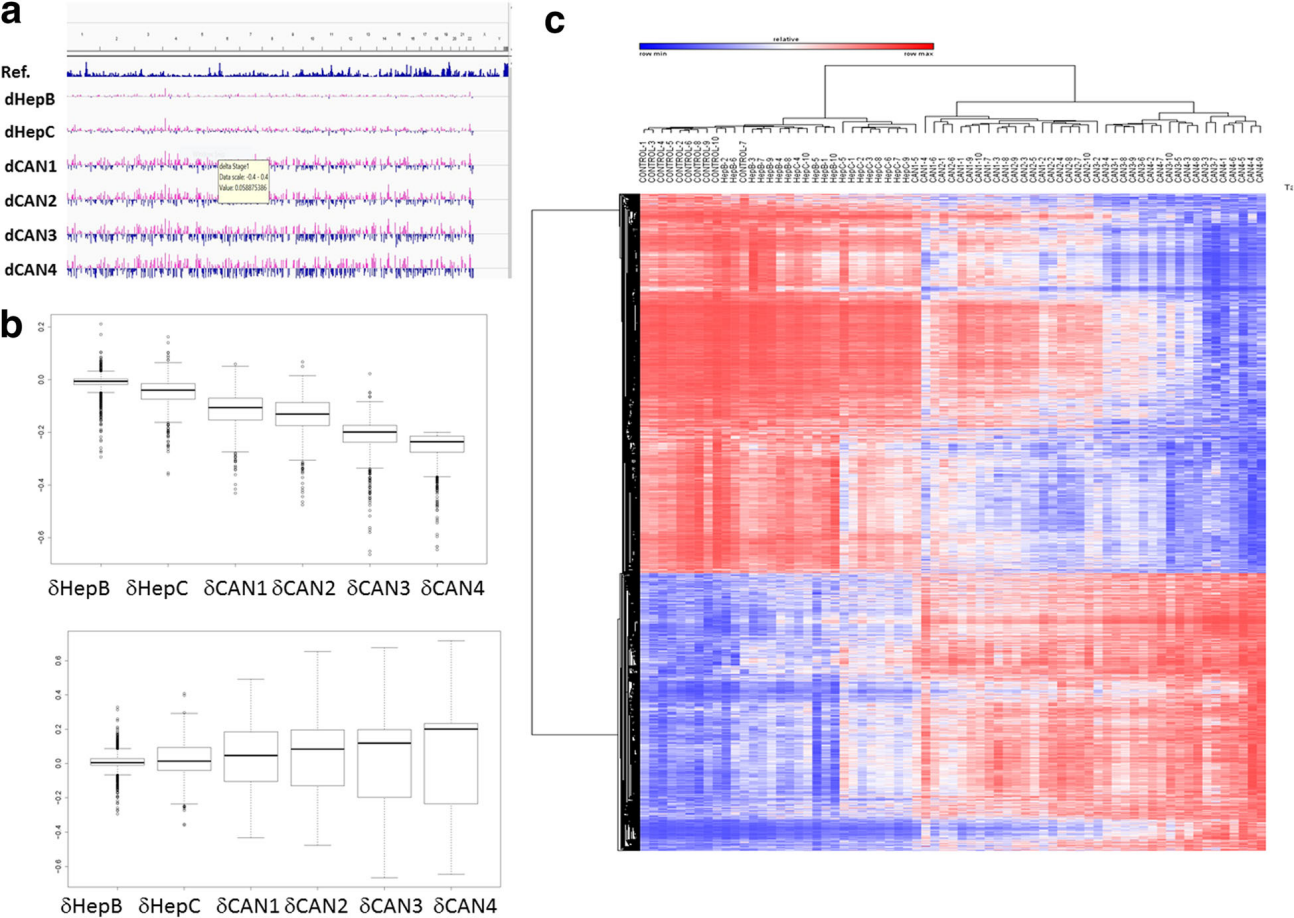
Correlation between quantitative distribution of site-specific DNA methylation levels and progression of HCC. **a** A genome wide view (IGV genome browser) of the escalating differences in DNA methylation from healthy controls (delta beta) in 3924 CG sites whose quantitative levels of methylation correlate with HCC progression (*r* > 0.8, *r* < − 0.8; delta beta > 0.2, < − 0.2; *p* < 10^−7^) in PBMC from HCC and hepatitis B and C patients. HepB-Hepatitis B; HepC-Hepatitis C; CAN1-stage 1 HCC; CAN2- Stage 2 HCC: CAN3- Stage 3 HCC; CAN4-Stage 4 HCC. **b** Box plot of DNA methylation delta beta values of the 3924 CG sites whose levels of methylation correlate significantly (*p* < 10^−7^) with HCC progression. Sites that are hypomethylated relative to healthy control during progression of HCC (upper panel) and sites that are hypermethylated relative to healthy controls (bottom panel) are shown separately. **c** Heat map of hierarchical clustering using one minus Pearson correlation of 69 people by DNA methylation beta values of the 3924 CG sites

Sixty-nine people from the Beijing area of China were included in a discovery set (10 controls and 10 patients for each of the following groups: hepatitis B and C, stages 1–3, and nine patients for stage 4) of HCC staged using the EASL–EORTC Clinical Practice Guidelines for HCC (Table 1 and “Methods” section). To address the question of whether quantitative differences in DNA methylation states in PBMCs correlate with progression of HCC (see “Methods” section for staging criteria) and whether this DNA methylation signature differentiates between chronic hepatitis B and C and HCC, we performed a genome-wide measurement of DNA methylation states in ~ 480,000 CpGs using the Illumina Infinium Human Methylation 450K BeadChip Array platform as described in the “Methods” section. Following normalization and batch correction, we performed a Pearson correlation analysis with Bonferroni correction for multiple testing (< 1 × 10^−7^) between the quantitative distribution of DNA methylation in the batch-normalized CGs across the array and progression of HCC. The analysis revealed a broad signature of DNA methylation that correlates with progression of HCC. A genome-wide view of the intensifying change in DNA methylation of 3924 robust differentially methylated sites (*r* > 0.8; *r* < − 0.8; delta beta > 0.2, > − 0.2, *p* < 10^−7^; Additional file 1: Table S1) during HCC progression is shown in Fig. 1a, b; notably, hypomethylation increases with progression of HCC (Fig 1b). The heat map in Fig. 1c shows the increasing differences in DNA methylation as HCC progresses and the clustering of all individual HCC patients away from healthy controls and hepatitis B and C individuals (except CAN1-5 who is clustered at the boundary between HepC and HCC (Fig. 1c). Importantly, PBMC DNA methylation profiles differentiate individual HCC patients at the earliest stage from hepatitis B and C, a critical challenge in early diagnosis of HCC.

**Table 1:** Clinical characteristics of the training set

Variable	Control (*n* = 10)	HepB (*n* = 10)	HepC (*n* = 10)	HCC1 (*n* = 10)	HCC2 (*n* = 10)	HCC3 (*n* = 10)	HCC4 (*n* = 9)	*p* value
Age (mean ± SD)	34.4 ± 8.5	36.9 ± 9.04	37.7 ± 12.08	52.9 ± 5.97	52.2 ± 12.2	56.1 ± 5.5	48 ± 15.06	5 × 10^−6^
Sex								0.034
Male	3 (30%)	8 (80%)	7 (70%)	9 (90%)	8 (80%)	9 (90%)	7 (70%)	
Female	7	2	3	1	2	1	2	
Alcohol								N.S.
No	8 (80%)	6 (60%)	8 (80%)	4 (40%)	5 (50%)	4 (40%)	5 (50%)	
Infrequent	2	1	2	2	1	0	2	
Heavy	0	3	0	4	4	6	2	
Smoking								N.S.
No	8 (80%)	5 (50%)	7 (70%)	6 (60%)	5 (50%)	4 (40%)	6 (60%)	
Quit	0	0	0	0	3	2	1	
Low	0	0	0	0	1	0	0	
Heavy	2	5	3	4	1	4	2	
Cirrhosis	0	0	0	0	0	0	0	
Hepatitis B	0	10	0	10	9	10	8	2 × 10^−16^
Hepatitis C	0	0	10	2	1	0		3 × 10^−16^
AFP (> 500u/ng)	0	1 (10%)	0	0	1 (10%)	3 (30%)	5 (50%)	0.043

*AFP* alpha feto protein, *HBV* Hepatitis B virus, HCV Hepatitis C virus

### Unique and overlapping differentially methylated sites associate with different HCC stages and differentiate HCC from hepatitis B and C; cross validation across HCC samples

**Fig. 2:**
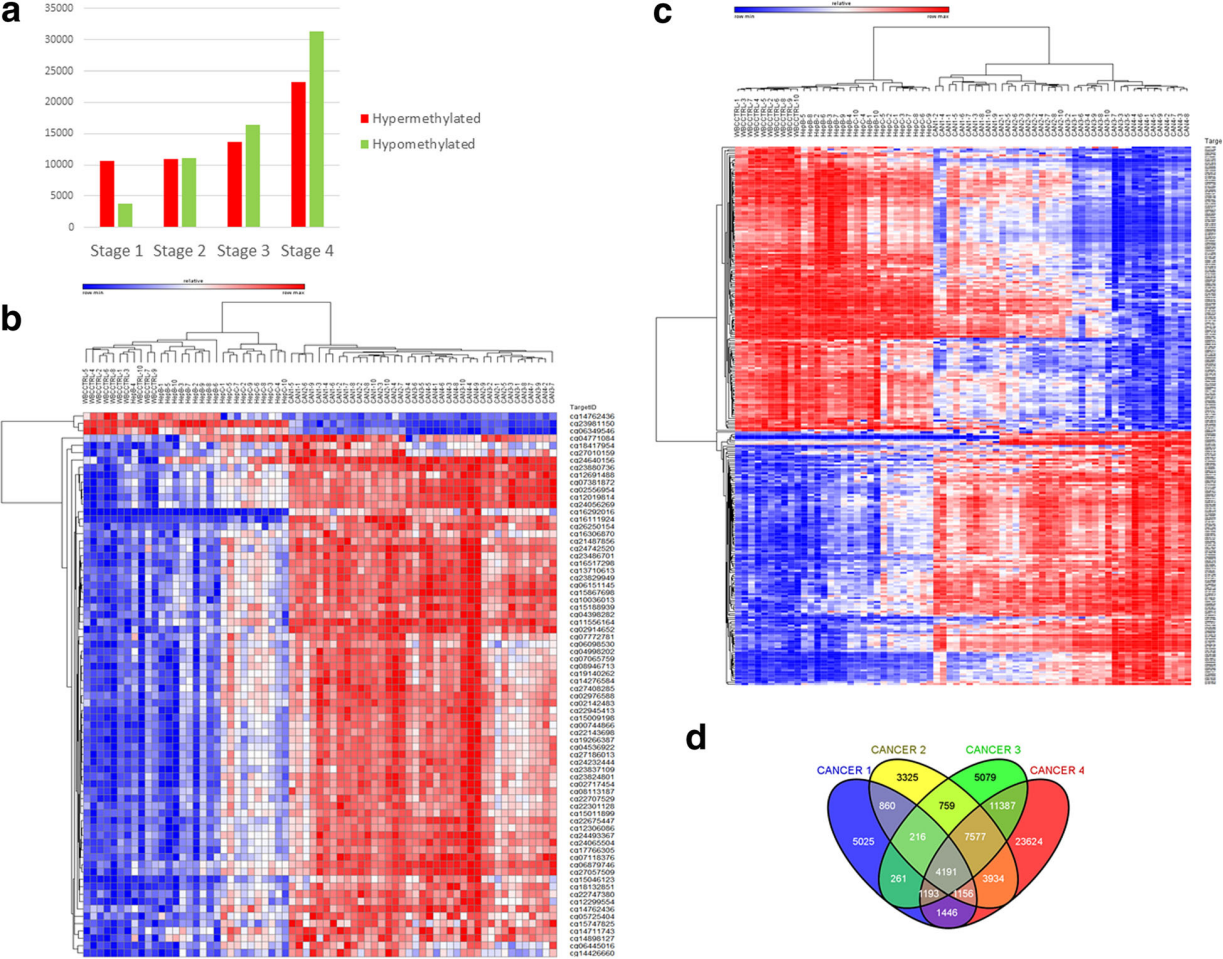
Differentially methylated CG sites at different stages of HCC and “cross-validation.” **a** Number of CG sites that are differentially methylated between different stages of HCC and healthy controls (*p* < 10^−7^) green: hypomethylated, red: hypermethylated. **b** Heat map presentation of hierarchical clustering of 69 people by 74 differentially methylated CGs between HCC stage 1 and control. **c** Heat map of hierarchical clustering of 69 people by 298 differentially methylated CGs between HCC stage 4 and control. **d** Ven diagram of the overlap between differentially methylated CG sites at different HCC stages (1–4). Significance was determined using Fisher exact test for all overlaps and all overlaps were highly significant (*p* < 1.7 × 10^−321^)

We delineated differentially methylated CGs between healthy controls and each of the HCC stages independently using the Bioconductor package Limma [44], [45] as implemented in ChAMP [46]. The number of differentially methylated CG sites (*p* < 1 × 10^−7^) between each stage of HCC and healthy controls increases with advance in stage; 14,375 for stage 1; 22,018 for stage 2; 30,709 for stage 3; and 54,580 for stage 4 (Additional file 2: Table S2, Additional file 3: Table S3, Additional file 4: Table S4 and Additional file 5: Table S5) with a notable increase in the fraction of hypomethylated sites (26% in stage 1 to 57% in stage 4) (Fig. 2a) as observed in the Pearson correlation analysis (Fig. 1b). We derived for each HCC stage a set of highly robust CG methylation markers (*p* < 1 × 10^−7^, delta beta > ± 0.3; < − 0.3 for HCC stage 1 and *p* < 10^−10^ delta beta of ± 0.3; for stage 2–4, we used a more stringent threshold for later stages to reduce the number of sites) (74 for stage 1, 14 for stage 2, 58 for stage 3, and 298 for stage 4). By combining the lists of markers derived independently for each stage and removing redundant CG sites between stages, we derived a combined non-redundant list of 350 CGs (Additional file 6: Table S6).

We used two methods of “cross-validation.” In the first method, all samples from one stage were used for “training” comparing the HCC samples to healthy controls. Subsequently, we tested the ability of the obtained signature to classify other stages and differentiate them from chronic hepatitis B and C, which were not “trained” for the said “CGs.” In the second method, samples of each group were randomized to two subsets, a “training set” and a “validation set,” and the signature of 369 significant CG sites obtained for the training set was tested on the “validation” set (Additional file 7: Figure S2).

Using the first method, the differentially methylated sites for each of the stages were derived by comparing ten healthy control and ten stage-specific HCCs. HCC of other stages and hepatitis B and C samples were not “trained” for these differentially methylated CGs and could serve as “cross-validation sets” to determine whether markers “trained” on one stage of HCC cluster correctly other HCC samples and whether they also differentiate HCC from other hepatitis B and hepatitis C.

As seen in Fig. 2b, c (stage 1 and 4) and Additional file 7: Figure S1 (stage 2 and 3), each of the independently derived set of markers for specific stages of HCC was “cross-validated” by its ability to cluster with a sharp boundary, all other HCC stages separately from controls, and hepatitis B and C samples that were not “trained” for these CGs. Interestingly, these markers also cluster hepatitis C and B samples separately from each other.

The overlap between independently derived CG markers that differentiate each of the HCC stages from healthy controls (Fig. 2d) is highly significant for all possible overlaps between the stages (hypergeometric test, *p* < 1.921718e-^319^) allowing for using of these differentially methylated CGs as peripheral markers of HCC.

**Fig. 3:**
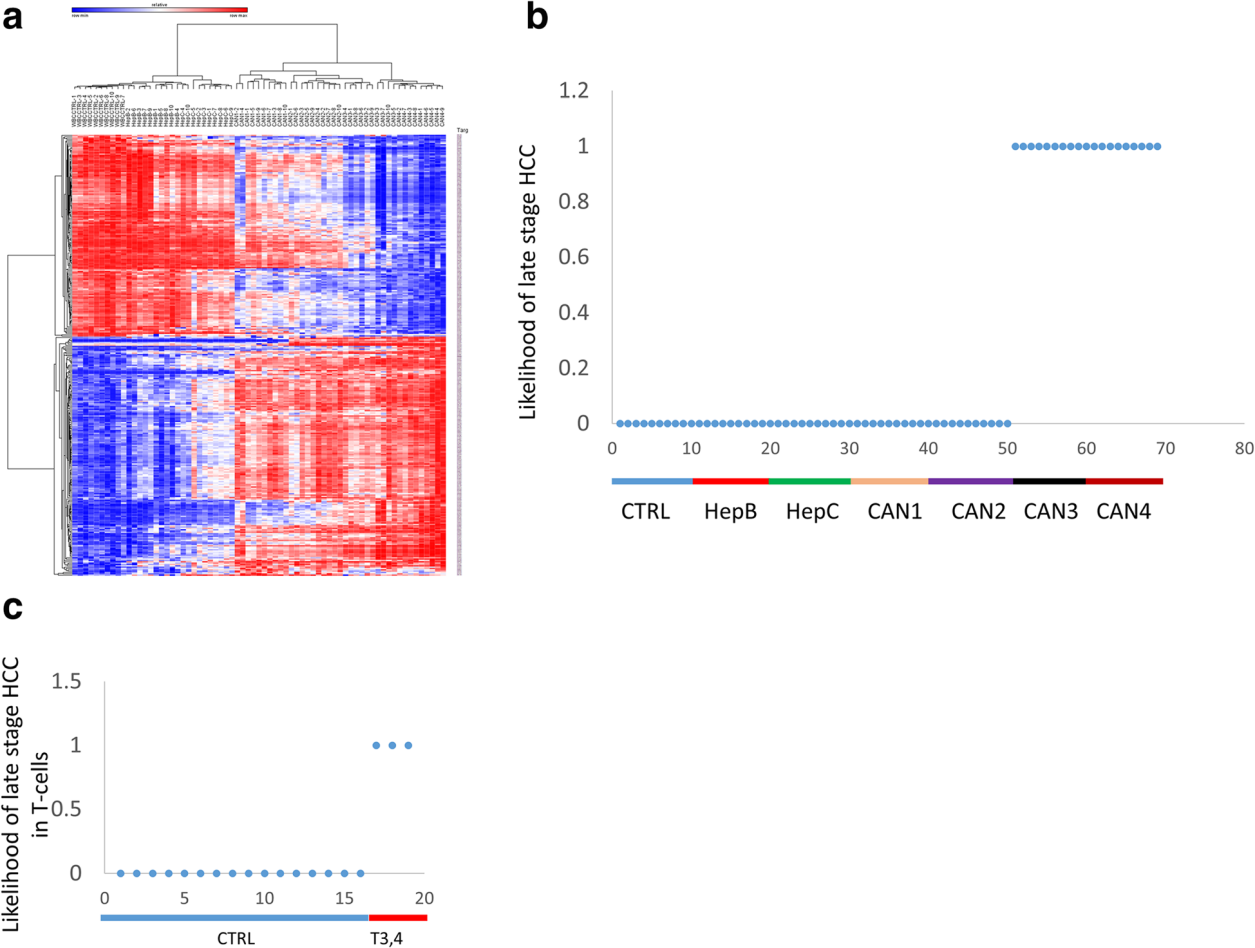
Staging of HCC using differentially methylated CGs. **a** Heat map presentation of hierarchical clustering of 69 people by 350 non-redundant CGs that are differentially methylated between different HCC stages and healthy controls. **b** Prediction of late stage HCC in 69 patients using a penalized model trained on a randomized half of the HCC patients and controls (“training set”) and tested on the other half (“validation set”). The plot shows all samples (the training and validation sets combined). The *y* axis indicates the predicted probability of late stage HCC for each person (from 0 to 1). **c** Prediction of late stages of HCC using the penalized model in T cell samples

We tested whether we could use the 350 CG list (described above) (Additional file 6: Table S6) to differentiate HCC stages from each other. Hierarchical clustering by one minus Pearson correlation of all samples using these 350 CGs correctly clustered the HCC samples by stage, and hepatitis B and C were clustered with healthy controls even though they were not “trained” by these CGs (Fig. 3a).

Since the 350 CG signature that was used to classify HCC stages was obtained by combining the signatures obtained for each stage, the signature has already been “trained” with the data used for testing. We therefore used a second method to “train” and “validate” a DNA methylation profile that classifies HCC stages. First, we randomly split each group (CTRL, HepB and C, and the different HCC stages) to two sets, a “training set” and a “validation set.” We then performed a correlation analysis between progression of HCC and levels of CG methylation. We selected the top 369 CGs (delta beta Can4-Can1 > 0.4, > − 0.4, adjusted *p* value < 0.05) (Additional file 7: Figure S2a left panel; Additional file 6: Table S6). Hierarchical clustering by one minus Pearson correlation of the “validation set” using these 369 CGs (trained in the “training set”) correctly clustered these other untrained HCC samples by stage while hepatitis B and C were clustered with healthy controls (Additional file 7: Figure S2a right panel). A randomized set of 369 CGs was unable to reveal the progressive alteration of the DNA methylation profile with advance of HCC stages (Additional file 7: Figure S2b).

To test whether we could delineate within the 350 CGs a shortlist of CG sites that differentiate early (stages 1 and 2) from late stages of HCC (stages 3 and 4), we performed a penalized regression on the “training set” that included randomized samples (five per group) from all HCC stages and all controls on the 350 CG list (Additional file 6: Table S6) using the R package “penalized” [47] which performs likelihood cross-validation and makes predictions on each left-out subject. The fitted model identified seven CGs (Additional file 8: Table S7) whose combined coefficients predicted with 100% accuracy the likelihood of stage HCC 3 and 4 cases and 100% specificity in calling HCC stage 1 and 2 as well as all controls (healthy and hepatitis B and C) as false. The penalized model was then used on the “validation set” of samples of HCC cases and controls to predict likelihood of each case being late stage HCC (Fig. 3b). We included in the test in addition to the new PBMC samples ten samples of T cells from healthy controls and ten T cell samples from different stages of HCC (Fig. 3c). Importantly, neither the 350 CG sites “classifier” nor the “penalized” model was previously “trained” with the T cell data. The penalized model predicted all the late stage samples including three late-stage HCCs in the T cells samples with 100% sensitivity and 100% specificity.

However, since the 350 CG signature that was used to classify HCC stages was obtained by combining the signatures obtained for each stage and has already been “trained” with the data used for testing, we also used the list of 369 CGs obtained from a “training set” that included representative samples from all cases and controls. We then performed a penalized regression on this set to identify CG sites that differentiate early (stages 1, 2) from late HCC (stages 3, 4). The fitted model identified a different set of 15 CGs (Additional file 8: Table S7) whose combined coefficients predicted with 100% accuracy the likelihood of stage HCC 3 and 4 cases and 100% specificity in calling HCC stage 1 and 2 as well as all controls (healthy and hepatitis B and C) as false. The penalized model was then used on the “validation set” of other samples of HCC cases and controls that were not used in training of either the selection of the 369 sites or the penalized model, to predict likelihood of each case being late stage HCC (Additional file 7: Figure S3). The penalized model predicted all the late stage samples with 100% sensitivity and 100% specificity. In summary, these data suggest that DNA methylation measurements could predict and differentiate HCC from controls and chronic hepatitis as well as early stage HCC.

### The DNA methylation signature of HCC remains significant after correction for potential confounders: sex, age, alcohol, smoking, and cell count

HCC patients in our study and in clinical setting are a heterogeneous group with respect to alcohol, smoking [48–51], sex [52], and age [53], and each of these factors are known to affect DNA methylation. In addition, white blood cells are a heterogeneous mixture of cells and alterations in white cell distribution between individuals might affect DNA methylation as well. We first determined the cell count distribution for each case using the Houseman algorithm [54]. Two-way ANOVA followed by pairwise comparisons and correction for multiple testing found no significant difference in cell count between the groups. We then performed a multivariate linear regression on the normalized beta values of the 350 CG sites that differentiate HCC from all other groups using group (HCC versus non HCC), sex, alcohol, smoking, age, and cell-count as covariates. All CG sites remained highly significant for group covariate even after including the other covariates in the model. Following Bonferroni corrections for 350 measurements, 342 CG sites remained highly significant for the group (HCC versus non HCC) (Additional file 9: Table S8). We performed a multifactorial ANOVA analysis on the beta values of the 350 sites as dependent variables and group (HCC versus non-HCC), sex, and age as independent variables to determine whether there are possible interactions between either sex and group age and group and between sex + age and group on DNA methylation. While the group remained significant for all 350 CGs, no significant interactions with sex or age were found after Bonferroni corrections (Additional file 10: Table S9).

### Differences in DNA methylation between HCC and healthy controls in T cells DNA overlap with differences in methylation in PBMC

**Fig. 4:**
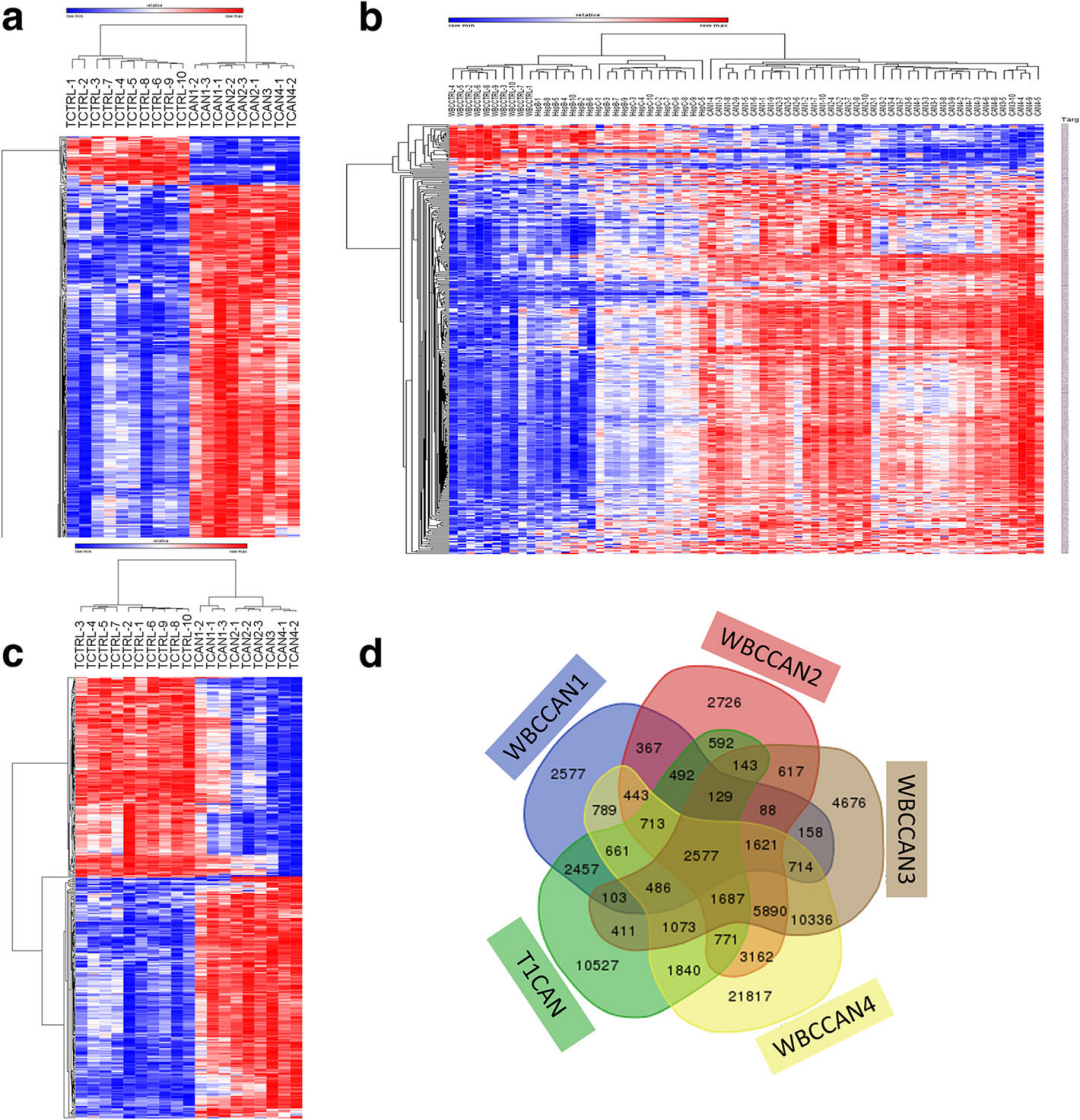
Differences in DNA methylation between HCC and healthy controls in T cells DNA overlap with differences in methylation in PBMC. **a** Heat map presentation of hierarchical clustering of ten healthy controls and HCC samples from T cells by 370 significantly differentially methylated CGs. **b** Heat map presentation of hierarchical clustering of PBMC DNA methylation samples from 69 people by 370 CGs “trained” in T cells. **c** Heat map presentation of hierarchical clustering of T cell samples from ten healthy controls and ten HCC by 350 CGs “trained” on T cell DNA. **d** Overlap of differentially methylated CGs in HCC in T cells and differentially methylated CGs in PBMC at different stages of HCC

Our multivariate analysis suggests that differences in PBMC DNA methylation between HCC and other groups (control and chronic Hepatitis) remain even when differences in cell count are taken into account. Furthermore, to determine whether differences in DNA methylation between cancer and control would disappear once the complexity of cell composition is reduced (although heterogeneity in cell subtypes remains), we analyzed the differences in DNA methylation profiles between T cells isolated from 10 of the 39 HCC patients included in the study (marked in Table 1) and all healthy controls (*n* = 10); the analysis revealed 24,863 differentially methylated sites at a threshold of *p* < 1 × 10^−7^ (Additional file 11: Table S10). Three hundred seventy robust sites (*p* < 1 × 10^−7^ and delta beta > 0.3, < − 0.3) correctly cluster all individual samples into two groups: HCC and controls (Fig. 4a) as well as cluster correctly all PBMC samples (*n* = 69) (Fig. 4b). The clustering analysis presented in Fig. 4b shows that CG sites that are differentially methylated in T cell DNA cluster individual HCC, hepatitis, and healthy control DNA samples from white blood cells with 100% accuracy. Thus, the differentially methylated CGs discovered using T cell DNA were cross-validated on different samples (29 different patients with HCC and 20 with chronic hepatitis) of PBMC DNA that were not used in training these CGs. Conversely, the 350 CGs that were derived by analysis of PBMC DNA from cancer stages and controls clustered the T cell healthy controls and HCC samples correctly (Fig 4c). There is a highly significant (hypergeometric test *p* = 0) overlap between the significant CGs (*p* < 1 × 10^−7^) that differentiate healthy controls from HCC using T cell DNA and CGs that differentiate the different HCC stages and controls using PBMC DNA (Fig. 4d). These data support the hypothesis that the differences in DNA methylation between HCC and other samples remain even when the complexity of cell types is reduced by isolation of particular cell types and provides further “cross-validation” for the association of these CGs with HCC.

### Differentially methylated genes in PBMC in HCC are enriched in immune-related canonical pathways

**Fig. 5:**
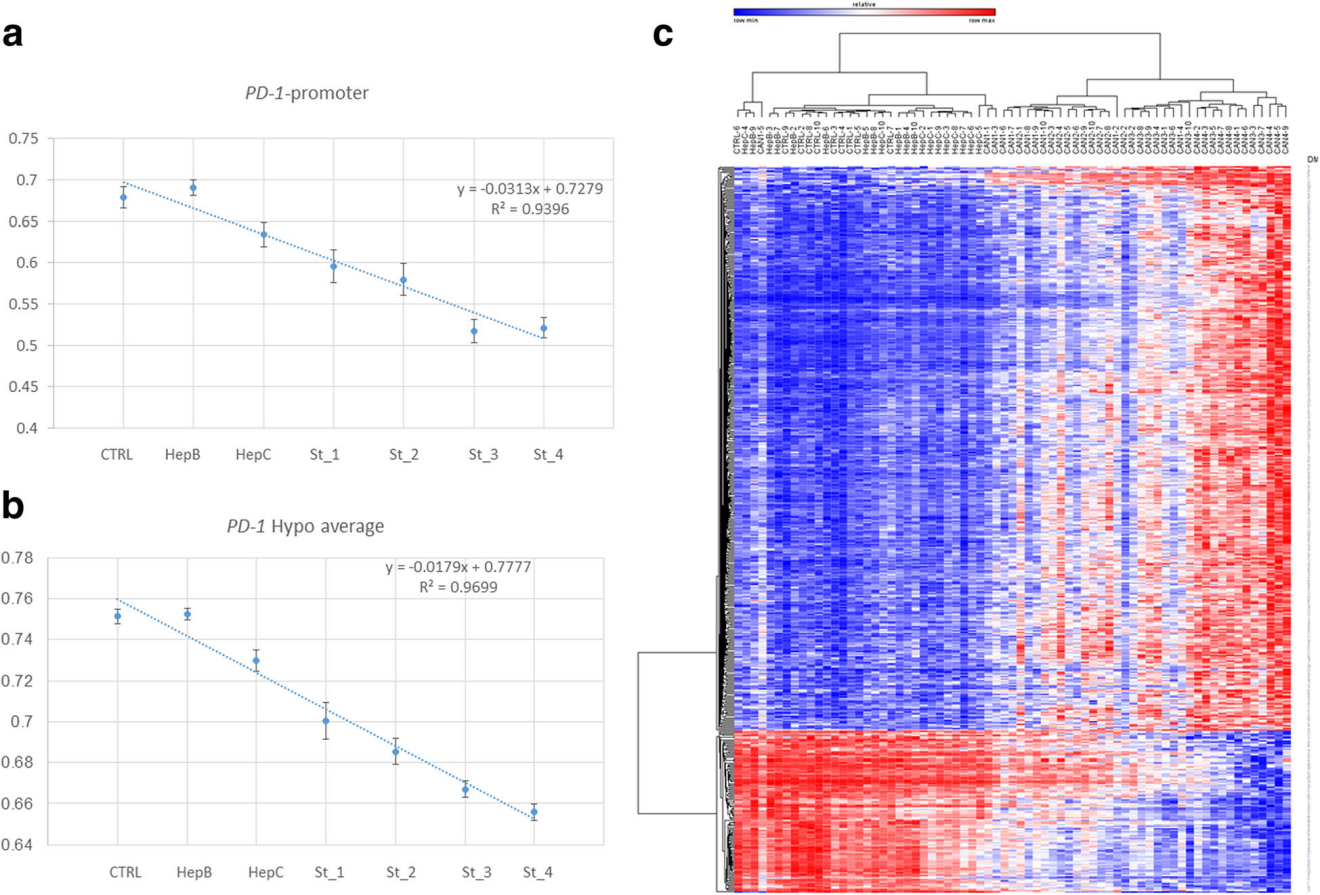
Progressive hypomethylation of PD-1 gene during HCC progression. **a** Correlation (linear fit) between average beta values for cg14453145 positioned at the TSS region of *PD-1* and control (CTRL) (stage code 0), hepatitis B (HepB) (1), hepatitis C (HepC) (2), and the four stages of HCC (St_1 to St_4) (3–6) diagnoses (equation and R values are indicated). One way ANOVA showed a highly significant effect of diagnosis on DNA methylation (*p* = 1 × 10^−13^; *F* = 20.77). Bonferroni adjusted pairwise comparison revealed significant differences between HCC stage 1 and control (*p* = 0.0058) and hepatitis B (*p* = 0.00079); between stage 2 and control (*p* = 0.0004) and hepatitis B (*p* = 4.9 × 10–^5^); between stage 3 and control (*p* = 4.8 × 10–^9^), hepatitis B (p = 4.9 × 10–^10^), hepatitis C (*p* = 1.8 × 10–^5^) and stage 1(*p* = 0.00993); between stage 4 and control (*p* = 2.1 × 10–^8^), hepatitis B (*p* = 2.3 × 10^−9^), hepatitis C (*p* = 5.9 × 10^−5^) and stage1 (*p* = 0.00558). **b** Correlation (linear fit) between average beta values of 24 CGs associated to the *PD-1* gene on the Illumina 450k arrays that were hypomethylated in HCC (average methylation score was calculated per person, the average of these scores were then calculated per group). There was a highly significant effect of diagnosis on DNA methylation as determined by one way ANOVA (*p* = 2.2 × 10^–16^, *F* = 52.74). Bonferroni adjusted pairwise comparison revealed significant differences between stage 1 HCC and control (*p* = 1.2 × 10^−7^), hepatitis B (*p* = 7 × 10^−8^), hepatitis C (*p* = 0.00487), stage 3 (*p* = 0.00081), and Stage 4 (*p* = 6.4 × 10^−6^); between stage 2 and control (*p* = 4.7 × 10^−11^), hepatitis B (*p* = 2.8 × 10^−11^), hepatitis C (*p* = 3.8 × 10^−6^), and stage 4 (*p* = 0.00645); between stage3 and control (*p* = 3.4 × 10^−15^), hepatitis B (2.1 × 10^−15^), hepatitis C (2.1 × 10^−10^), and stage 1 (p = 0.00081); and between stage 4 and control (2 × 10^−16^), hepatitis B (2 × 10^−16^), hepatitis C (1.8 × 10^−12^), stage 1 (6.4 × 10^−6^), and Stage 2 (*p* = 0.00645). **c** Heat map presentation of hierarchical clustering (city block) of 69 people by 5’DMR whose average methylation correlates with HCC progression

Progression of HCC has a broad footprint in the methylome (Fig. 1a). To gain insight into the functional footprint of the differentially methylated genes in PBMC and T cells from HCC patients, the gene lists generated from the differential methylation analyses were subjected to a gene set enrichment analysis using Ingenuity Pathway Analysis (IPA). We first subjected genes associated with CGs that showed linear correlation with stages of HCC in the Pearson correlation analysis (Fig. 1b) (*r* > 0.8; *r* < − 0.8; delta beta > 0.2, < − 0.2). Notably, the top upstream regulators of genes associated with these CGs are TGFbeta (*p* < 1.09 × 10^−17^), TNF (*p* < 7.32 × 10^−15^), dexamethasone (*p* < 7.74 × 10^−12^), and estradiol (*p* < 4 × 10^−12^) which are major immune, inflammation, and stress regulators of the immune system. Top diseases identified were cancer (*p* value 1 × 10^−5^ to 2 × 10^−51^) and hepatic disease (*p* < 1.24 × 10^−5^ to 1.11 × 10^−25^). A strong signal was noted for liver hyperplasia (*p* < 6.19 × 10^−1^ to 1.11 × 10^−25^) and hepatocellular carcinoma (*p* < 5.2 × 10^−1^ to 3.76 × 10^−25^). An inspection of the genes that are differentially methylated reveals a large representation of immune regulatory molecules such as *IL2*, *IL4*, *IL5*, *IL16*, IL7, *Il10*, *IL18*, *Il24*, *Il1B* and interleukin receptors such as *IL12RB2*, *IL1B*, *IL1R1*, *IL1R2*, *IL2RA*, *IL4R*, *IL5RA*; chemokines such as *CCL1*, *CCL7*, *CCL18*, *CCL24*, as well as chemokine receptors such *CCR6*, *CCR7* and *CCR9*; cellular receptors such as *CD2*, *CD6*, *CD14*, *CD38*, *CD44*, *CD80* and *CD83*; *TGFbeta3* and *TGFbetaI*, *NFKB*, *STAT1*, *STAT3* and *TNFa*. Notably, a CG site in the promoter of PD-1, a protein that triggers an immune checkpoint and is now recognized as promising clinical target for anti-immune-blockade cancer treatment [43], is gradually demethylated in PBMC as HCC progresses (Fig. 5a). Differential methylation of 29 out of 78 CG probes associated with PD1 in the 450K array strongly correlated with HCC progression (*R* < − 0.7, *Q* < 1 × 10^−8^), a highly significant enrichment (hypergeometric test, *p* = 4.3 × 10^−238^) (Additional file 12: Table S11). The average methylation of all significantly hypomethylated *PD-1* CGs was significantly correlated with HCC progression (Pearson cor *R* = − 0.9, *p* < 1.7 × 10^−321^) (Fig. 5b).

A comparative IPA analysis between differentially methylated genes in PBMC and T cells revealed *NFKB*, *TNF*, *VEGF* and *IL4* and *NFAT* as common upstream regulators. Overall, the DNA methylation alterations in HCC PBMC and T cell show a strong signature in immune modulation functions and are consistent with the emerging role of the immune system in cancer. We have previously delineated differentially methylated promoters between HCC biopsies and noncancerous liver tissue [23]. We found a nonsignificant overlap (*n* = 44) (hypergeometric test; *p* = 0.76) between promoters that are differentially methylated in HCC in the cancer biopsies (1983) and PBMC (545) (*p* < 1 × 10–7; delta beta < − 0.2; > 0.2). These data support the hypothesis that changes in DNA methylation seen in PBMC reflect changes in the immune system in HCC and are not a footprint of circulating DNA from tumors or tumor surrogates.

Since methylation of individual CGs across regions are never homogenous, we analyzed extended differentially methylated regions (DMRs) between all cancers and all controls including chronic hepatitis B and C using the champ.lasso function in Champ [55], shortlisted DMRs in 5′ regions of genes, and calculated their average methylation levels. We identified 4261 significant DMR (FDR adjusted *p* < 0.05) in promoters and 5′ upstream regions. We then tested whether the average methylation levels of these promoter DMRs correlate with cancer progression using Pearson correlation analysis. Five hundred thirty DMRs ranging from 19 to 8951 bases showed highly significant correlation with HCC progression (*r* > 0.8; *r* < − 0.8, *p* = 0) (Additional file 13: Table S12) suggesting that differential methylation during HCC progression involves broad regulatory regions and is not limited to scattered individual CG sites. These DMRs clearly cluster all HCC away from all controls including hepatitis B and C and nicely differentiate HCC from hepatitis (Fig. 5c).

### Validation of differentially methylated CGs by pyrosequencing

**Fig. 6:**
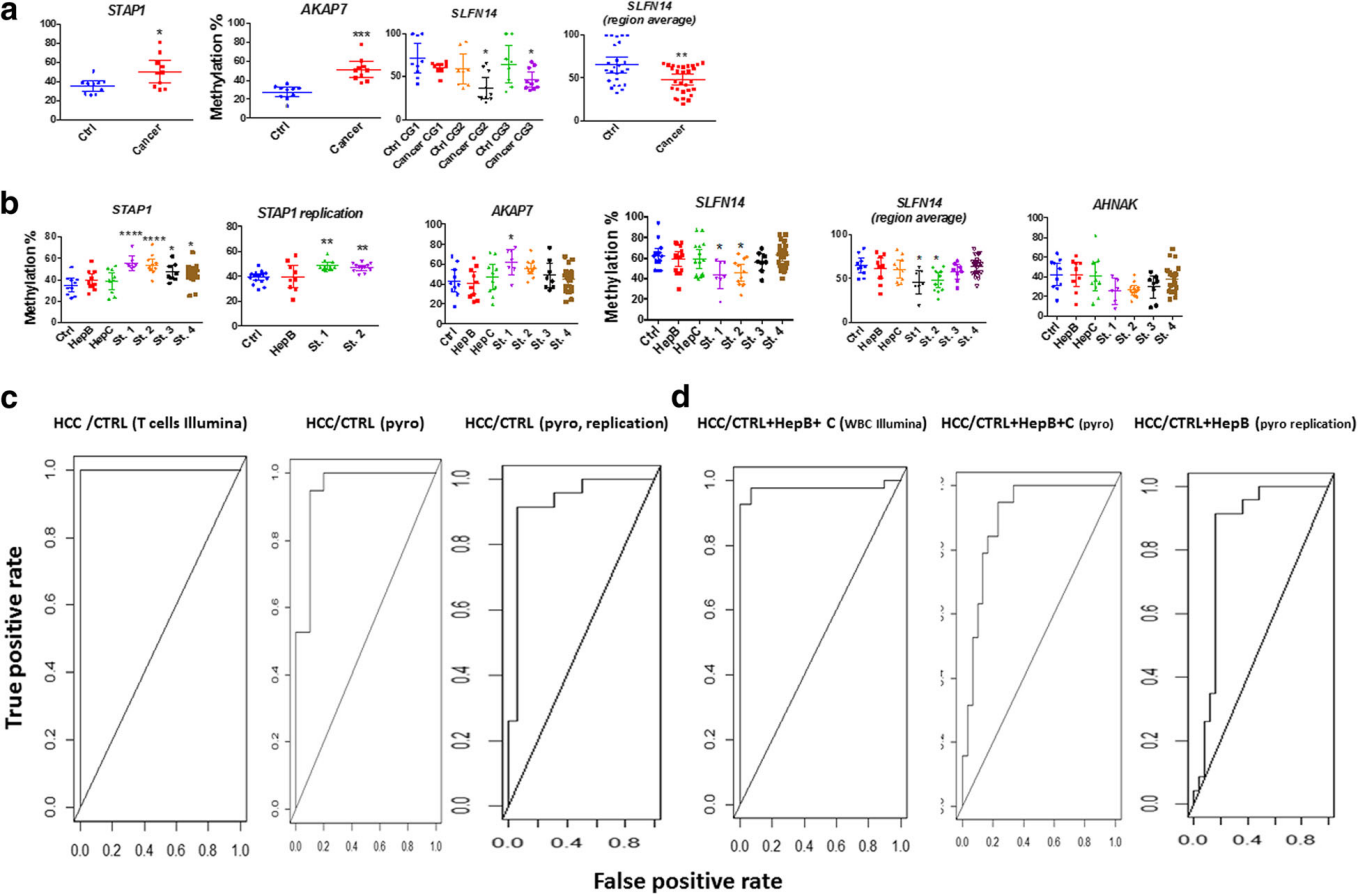
Validation of differentially methylated CGs in the discovery set and validation set by pyrosequencing. **a** Top row, CG sites that are differentially methylated between HCC (*n* = 10) and healthy controls (*n* = 10) in T cells (significance was measured by student *t* test set at a threshold of < 0.05). The primers for pyrosequencing and conditions are listed in Additional file 18: Table S17. The scattered plot shows the mean and 95% confidence intervals (C.I.). The average methylation for three CG sites in the SLFN14 differentially methylated region is shown in the left panel. Summary of statistics including CI, SD, and SEM values are presented in Additional file 16: Table S15. **b** Validation by pyrosequencing of DNA extracted from T cells in the validation set. ANOVA was used to compare variance between the hepatitis B (HepB) control and other groups healthy (*n* = 10), hepatitis B (*n* = 10), hepatitis C (HepC) (*n* = 10) group and the HCC stages 1 (*n* = 8), 2 (*n* = 12), 3 (*n* = 8), and 4 (*n* = 22). *STAP1* replication presents pyrosequencing data from T cells DNA from the second replication cohort (Additional file 15: Table S14). **c** ROC curve measuring specificity (*Y* axis) and sensitivity (*X* axis) of *STAP1* methylation as a biomarker for discriminating HCC from healthy controls in T cells first cohort (Illumina 450 K data), in first validation set (pyrosequencing) and third validation set (pyrosequencing replication). **d.** ROC curve for *STAP1* methylation as a biomarker for distinguishing HCC from healthy persons and chronic hepatitis in PBMC (Illumina), first validation set (pyrosequencing), and third validation set (pyrosequencing, replication). Statistic code: * 0.05, ** 0.01, *** 0.001

We randomly selected CG sites that were significantly different between HCC and controls in T cells that were either hypermethylated *A Kinase* (*PRKA*) *Anchor protein 7* (*AKAP7*) gene, the *Signal Transducing Adaptor Family 1* (*STAP1*), or hypomethylated the *Schlafen family member 14*(*SLFN14*) gene for validation using pyrosequencing (Fig. 6a). The *SLFN14* region that we validated contained three CGs which allowed us to calculate the average methylation of the region which is also significantly different between HCC and controls (Fig. 6a).

For our validation set, we used T cells DNA to reduce cell composition issues. The validation set included 80 people, ten healthy controls and ten individuals from each of the hepatitis B and C (total control *n* = 30) and 50 HCC (HCC stage1 *n* = 8; stage 2 *n* = 12, stage 3 *n* = 11 and stage 4 *n* = 19) (Table 2 and Additional file 14: Table S13) and examined the same genes as well as one additional hypomethylated gene: *Neuroblast differentiation-associated protein (AHNAK)* (cg14171514) (6b). Linear regression between all controls including chronic hepatitis B and C (healthy and hepatitis B and C) and HCC stages 1 and 2 (0+A) revealed significant association with early HCC stages (1, 2) for all four CGs after correction for multiple testing (*STAP1 p* = 4.04 × 10^−7^; *AKAP7 p* = .0.046; *SLFNL14 p* = 0.012; *AHNAK p* = 0.003436. Linear regression between all controls and all stages of HCC revealed significant association for *STAP1* (*p* = 1.6 × 10^−6^) and *AHNAK* (*p* = 0.026) with HCC after correction for multiple testing. One way ANOVA analysis was performed with methylation as the dependent variable, and the seven diagnosis groups (healthy, hepatitis B, hepatitis C, HCC stage 1 to 4) as independent variables revealed significant effect for diagnosis (*F =* 7.263; *p <* 7.49 *×* 10^−6^) on *STAP1*methylation. Dunnett test, a multiple comparison procedure which compares each of the number of treatments with a single control, revealed significant differences between HCC stage 1 (BCLC 0) and HepB (*p < 0.01*) and stage 2 and HepB (*p < 0.01*) and no significant difference between chronic hepatitis B and healthy controls, hepatitis B and hepatitis C, and hepatitis B and late stages HCC. There was a significant effect of diagnosis on *AKAP 7* methylation (*F =* 2.71155; *p =* 0.0198). A multiple comparison test (Dunnett) between the different diagnosis groups and hepatitis B revealed significant differences between HCC stage 1 (BCLC 0) and hepatitis B (*p <* 0.05), but not between either stage 2 and hepatitis B, hepatitis B and healthy controls, hepatitis B and hepatitis C, and hepatitis B and late stages HCC. There was a significant effect of diagnosis on *SLFN14* methylation (Cg00974761) (*F =* 3.877; *p =* 0.0018). A multiple comparison test (Dunnett) between the different diagnosis groups and hepatitis B revealed significant differences between HCC stage 1 (BCLC 0) and hepatitis B (*p* < 0.05), but not between either stage 2 and hepatitis B, hepatitis B and healthy controls, hepatitis B and hepatitis C, and hepatitis B and late stage HCC. We also measured the average methylation of three CG sites in the *SLFN14* differentially methylated region. There was a significant effect of diagnosis on DNA methylation of this region (*F =* 3.727, *p =* 0.0028). A multiple comparison test (Dunnett) between the different diagnosis groups and hepatitis B revealed significant differences between early stages HCC stage 1+2 and controls when they were combined (*p* < 0.05), but no other significant differences were detected in the other pairwise comparisons. There was a significant effect of diagnosis on *AHNAK* methylation (*F =* 2.461; *p =* 0.0323). A multiple comparison test revealed significant differences between early stages HCC stage 1+2 and controls when they were combined (*p* < 0.05) but no other significant differences were detected in the other pairwise comparisons (Fig. 6b).

**Table 2:** Clinical characteristics of the validation set

Variable	Control (*n* = 10)	HepB (*n* = 10)	HepC (*n* = 10)	HCC1 (*n* = 8)	HCC2 (*n* = 12)	HCC3 (*n* = 11)	HCC4 (*n* = 19)	*p* value
Age (mean ± SD)	35.5 ± 7.5	43.9 ± 15.46	50.1 ± 14.7	61.75 ± 9.39	56 ± 7.45	48.09 ± 7.49	53.63 ± 9.77	5 × 10^−5^
Sex								
Male	6 (60%)	7 (70%)	5 (50%)	8 (100%)	10 (83%)	11 (100%)	16 (84%)	
Female	4	3	5	0	2	0	3	
Alcohol								8 × 10^−4^
No	NA	10 (100%)	9 (90%)	7 (87.5%)	8 (67%)	3 (27%)	9 (47%)	
Quit	NA	0	1	0	0	0	1	
Infrequent	NA	0	0	0	1	0	0	
Heavy	NA	0	0	1	3	8	9	
Smoking								0.035
No	NA	6 (60%)	8 80%)	4 (50%)	8 (67%)	9 (82%)	9 (47%)	
Quit	NA	1	0	0	1	0	0	
Low	NA	0	0	0	0	0	1	
Heavy	NA	3	2	4	3	2	9	
Cirrhosis	0	0	0	0	0	0	0	
Hepatitis B	0 (0%)	10 (100%)	1 (10%)	8 (100%)	12 (100%)	11 (100%)	19 (100%)	3 × 10^−13^
Hepatitis C	0 (0%)	0 (0%)	10 (0%)	0 (0%)	0 (0%)	0 (0%)	0 (0%)	0
AFP	0 (0%)	0 (0%)	0 (0%)	1 (12.5%)	2 (16.6%)	4 (36%)	9 (47.3%)	0.34

Since our validation test indicated that *STAP1* effectively discriminated between either healthy controls or chronic hepatitis and early stages of HCC, we further validated association of *STAP1* methylation with stages 1 and 2 HCC in T cell DNA derived from a third independent cohort (*n* = 48) of healthy controls (*n* = 16) HepB (9) and HCC stages 1 (*n* = 11) and 2 patients (*n* = 12) (Additional file 15: Table S14). We included HepB samples as a control since all HCC samples were HepB positive. Linear regression between all healthy controls and stages 1 and 2 revealed significant association with early HCC stages (1, 2) (*p* = 6.9 × 10^−7^, *F* = 35.62), which remained significant even when sex and age were included in the model as covariates (*p =* 1.9 × 10^−6^). Linear regression between all controls including hepatitis B and HCC stages 1 and 2 revealed a significant association (*p* = 3.3 × 10^−5^, *F* = 21.18) (Fig. 6b). We performed a multifactorial ANOVA analysis on the methylation values of *STAP1* as a dependent variable and diagnosis (HCC versus non-HCC including hepatitis B), sex, and age as independent variables as well as interactions between sex, age, and diagnosis. Our analysis revealed a significant main effect of diagnosis (*p* = 4.88 × 10^−5^, *F* = 20.48) and no significant interaction between sex and diagnosis (*p* = 0.96, *F* = 0.0024) or age and diagnosis (*p* = 0.829, *F* = 0.0473). There was no significant effect of either age (*p* = 0.1329, *F* = 2.349) or sex (*p* = 0.7529, *F* = 0.1004) on DNA methylation. One way ANOVA analysis performed with methylation as the dependent variable and the seven diagnosis groups (healthy, HepB, HCC stage 1 to 2) as independent variables revealed significant effect for diagnosis on *STAP1* methylation (*F* = 6.983, *p* = 0.0006). A multiple comparison test (Dunnett) between the different diagnosis groups and HepB (since all HCC patients were positive for HepB) revealed significant differences between HCC stage 1 and HepB (*p* < 0.01) and stage 2 and HepB (*p* < 0.05), but no difference was detected between HepB and healthy controls (Fig. 6b) (summary of ANOVA tests and descriptive statistics could be found in Additional file 16: Table S15).

### Diagnostic value of differentially methylated CGs in peripheral T cells and PBMC

A measure of the diagnostic value of a biomarker is the receiver operating characteristic (ROC) which measures sensitivity as a function of specificity and determines a threshold value for a predictor which provides the highest accuracy as a biomarker for differentiating case from control [56] (Fig. 6c, d). We first determined ROC characteristics for the normalized Illumina 450K beta values for T cells from healthy controls and HCC (Fig. 6c). *STAP1* (cg04398282) behaved as a potential biomarker (accuracy 100%, AUC 1 and both sensitivity and specificity 100%). The *STAP1* biomarker was discovered by comparing T cell DNA methylation from HCC and healthy controls (Additional file 11: Table S10). We therefore cross-validated the biomarker properties of STAP1 cg04398282 by examining the ROC characteristics using normalized beta values from the PBMC DNA samples which included hepatitis B and hepatitis C patients as well as 29 additional HCC patients that were not included in the T cell DNA methylation analysis (Fig. 6d). The accuracy is 96% using a threshold beta value of 0.6729; AUC was 0.9741379 (sensitivity 0.975 and specificity 0.973). We then examined the ROC characteristics using pyrosequencing values of *STAP1* in the validation set of T cell DNA (Fig. 6c). The accuracy of calling HCC 1,2 from all other controls (healthy and hepatitis B and C) is 85.7% using a threshold beta value of 0.50; AUC is 0.898 (89.5% sensitivity and 83% specificity) (Fig. 6d). In the third cohort of T cell DNA (Additional file 15: Table S14), the accuracy of calling HCC stages 1 and 2 from all other controls (healthy and hepatitis B) using a threshold of 44.5 is 87.5%; the AUC is 84.7% (91.3 sensitivity and 84% specificity). The accuracy of differentiating HCC stages 1 and 2 from healthy controls is 92.3%; the AUC is 0.924 (91.3% sensitivity and 93.75% specificity). We noted however a “batch effect” in pyrosequencing. While we can compare the groups within a study, overall methylation levels vary between experiments done at different times, which will require in the future a normalization procedure that will allow comparisons across different batches.

In summary, *STAP1* provides proof of principle for potential DNA methylation biomarkers in HCC peripheral white blood cells and for discriminating Stage 1 from chronic hepatitis and healthy controls which is a critical hurdle in early diagnosis of liver cancer. The other three CGs that we have validated using pyrosequencing to be associated with stage 1 HCC in the validation set do not exhibit biomarker properties in ROC curves. Further experiments are required to delineate and validate other high quality biomarkers from the list of associated DNA methylation sites that we have delineated in this paper.

## Discussion

The focus in DNA methylation studies in cancer to date has been on the tumor, tumor microenvironment [27, 28], and circulating tumor DNA [24, 25], and major advances were made in this respect. In this study, we focused however on the host immune system since the idea that the qualities of the host immune system might define the clinical emergence and trajectory of cancer has been proposed almost a century ago [2] and there is an emerging line of evidence that is consistent with this hypothesis [6–19]. New approaches to cancer therapy are targeted at boosting the host immune system [43]. HCC is a very interesting example since it frequently progresses from preexisting chronic hepatitis and liver cirrhosis [42] and could provide a tractable clinical paradigm for addressing this question.

Our analysis revealed a large number of sites whose quantitative state of methylation strongly correlates (*r* = ± 0.8–0.9) with progression of HCC which is consistent with the idea that DNA methylation alterations in the immune system are tightly linked with the development of HCC. Interestingly, the overall direction of the differences in DNA methylation changes as HCC advances, from hypermethylation to hypomethylation. Importantly, there is a sharp boundary between stage 1 HCC and chronic hepatitis B and C supporting the hypothesis that changes in DNA methylation are linked with the transition from chronic hepatitis B and C to HCC. It should be noted however that our study is a cross-sectional study and we cannot distinguish at this stage between progressive changes in the same individual from intrinsic differences between people who develop advanced cancer and those who have early stage HCC. Future longitudinal studies in the same patients will be required to address this important question.

Careful inspection of the DNA methylation profiles of chronic hepatitis and HCC in Fig. 1a suggests however that some of the CG sites that undergo large changes in methylation as HCC progresses are already slightly altered in chronic hepatitis. This is consistent with the fact that HCC often progresses from chronic hepatitis and suggests that the changes in DNA methylation in PBMC are seeded by chronic hepatitis and that they might be playing a role in the progression from chronic hepatitis to HCC. Nevertheless, the changes in DNA methylation between chronic hepatitis and early HCC are dramatic, and a clear boundary is seen in our heat maps between chronic hepatitis and HCC that might be utilized to diagnose early transition from chronic hepatitis to HCC.

We also used a “case-control” approach comparing each stage of HCC independently (*n* = 10) with healthy controls (*n* = 10). Cross validation revealed highly significant overlap between differentially methylated CGs in the four stages of HCC. DNA methylation data for chronic hepatitis B and C were not used for deriving the HCC-stage differentially methylated CGs; nevertheless, these CGs accurately clustered the hepatitis samples with the healthy controls separately from other HCC stages. Thus, these markers were “cross-validated” using a separate set of non-HCC samples and other HCC samples, further demonstrating clear boundary in DNA methylation profiles between all HCC samples and chronic hepatitis. Although differentially methylated CGs overlapped between different HCC stages, they nevertheless differentiated stages of cancer from each other because of the intensifying changes in DNA methylation at these sites with progression of HCC. Importantly, the differentially methylated sites remained significant even after taking into account in the regression model differences in sex, age, smoking, and alcohol abuse as well as differences in cell type distribution. Combined, these data support the conclusion that the emergence and progression of HCC is linked with robust and broad changes in DNA methylation in the peripheral immune system.

We further “cross-validated” this conclusion by examining DNA methylation profiles in isolated T cells from a subset of ten HCC patients and the ten healthy controls. If indeed changes in DNA methylation that were detected in PBMC were an artifact of differences in cellular distribution, they should “disappear” using an isolated subset of white blood cells. In addition, by using a different set of DNA samples analyzed independently, we could rule out technical and random effects. We show a robust signature of HCC in peripheral T cell DNA methylation that significantly overlaps with changes in DNA methylation in PBMC and correctly clusters other “untrained” HCC samples separately from chronic hepatitis using DNA methylation data from PBMC. Conversely, differentially methylated CG sites derived from comparing PBMC DNA methylation profiles accurately cluster HCC samples away from controls using T cell DNA methylation data.

Examination of the “gene set” that is differentially methylated in peripheral immune cells in HCC provides some cues as to the potential implications of these changes. It is important to note that in difference from circulating tumor DNA, differentially methylated CGs delineated in our study in PBMC and T cells do not overlap significantly with previously characterized differentially methylated genes in HCC tumors [23].

Analysis of the upstream regulators of the differentially methylated genes provides an overall picture of the functional pathways that are affected. The list includes members of nodal inflammatory and immune regulatory pathways such as TGFbeta, TNFalpha, and the glucocorticoid receptor. Notably, the upstream regulators TGFbeta and TNFalpha are differentially methylated themselves. Interestingly, the enrichment of stress responsive glucocorticoid-regulated pathway might indicate that a fraction of the changes in DNA methylation reflects activation of stress-related processes in the HCC patients. Differentially methylated genes include nodal transcription factors in the immune system and inflammatory response such as NFAT, STAT3, and NFKB; a rich representation of interleukins, chemokines, chemokine receptors and nodal cellular antigens that are involved in cellular fate and differentiation such as CD38 CD44 as well as PD-1 a protein that controls an immune response checkpoint and is now recognized as a promising clinical target for immunity-boosting anti-cancer treatment [43]. CD38 is an enzyme that synthesizes cyclic ADP-ribose and nicotinate-adenine dinucleotide phosphate, is expressed in leukocytes and functions in cell adhesion and calcium signaling. Interestingly, CD38 is associated with plasmablastic lymphoma and prolymphocytic leukemias. CD44 is a cell surface glycoprotein that is involved in multiple cellular functions relevant to the immune system including lymphocyte activation, recirculation and homing, hematopoiesis, and tumor metastasis. It is unclear how these broad and complex changes in methylation of genes in immune and inflammatory pathways in PBMC affect the overall output and function of the immune system and inflammatory reactions during progression of HCC. Our data provides however compelling data implicating an escalating epigenetic reprogramming of the immune and inflammatory systems during HCC advancement.

The relationship between changes in DNA methylation and steady state transcription is complex. Moreover, DNA methylation alterations might “program” genes in the immune system to respond to transient signals that are time and context dependent and are not captured by examining steady state mRNA levels. Extensive future experiments are required to understand how this “epigenetic” reprogramming of the immune system affects its function in promoting/suppressing HCC. One interesting question that remains to be answered is whether the changes described here for HCC occur in other cancers or whether these changes are unique to HCC, a disease that frequently emerges from chronic inflammation of the liver. However, the changes in DNA methylation in HCC are dramatically enhanced in comparison with the effects of chronic hepatitis on DNA methylation supporting the conclusion that these are cancer-related changes.

A cardinal question that our data is raising is whether these changes in DNA methylation are a cause or consequence of HCC. The fact that the changes in DNA methylation intensify with the advance of HCC is consistent with the idea that they respond to HCC progression. However, they might be still playing an important role in the escalation of the disease. This idea is consistent with recent approaches to cancer therapy that target the immune system to boost a cytotoxic T cell response to tumor cells such as current anti-PD-1 treatments [43]. It is tempting to speculate that targeting the epigenetic changes in the immune system in addition to the changes in the tumor itself might have therapeutic effects. Our data suggests that this possibility should at least be considered.

The observation that HCC has a DNA methylation signature in easily accessible PBMC and T cells points to the opportunity that these could serve as “noninvasive” biomarkers for detection of early transition from chronic hepatitis to HCC as well as HCC progression. We provide here several examples that present a “proof of principle” for using PBMC differentially methylated CGs as “biomarkers” in HCC. However, future extensive analyses of large samples of HCC and controls are required before it is possible to confirm that such “biomarkers” have sufficient accuracy to differentiate early stage HCC from controls. It is also expected that a single CG site will not have sufficient accuracy and that a combination of sites within a region will be required. Nevertheless, this study provides a “proof of principle” for further exploring this opportunity.

One limitation of our data is the relatively small number of samples. However, the effect sizes that we observe are large because of the low average variance in DNA methylation values across individuals. A power calculation using the pooled standard deviation of control and stage 1 HCC groups (0.022) and desired power of 0.8 shows that an extremely “small” (unreliable) sample size (*n* = 4) is required to detect a delta beta of 0.1 at genome-wide significance (*p* = 1 × 10^−7^). This large effect size might explain how sites that were discovered by comparing two groups with sample sizes of 10, cross-validated in other samples and were replicated. Power calculation suggests that increasing the sample size from 10 to 100 would not increase power as it is maximized with *n* = 10. However, it is clear that further replication is required to rule out random bias or stratification in our samples.

## Conclusions

Our study shows that the host immune system has a distinct DNA methylation signature in cancer, that this signature intensifies as cancer progresses, and that this signature differentiates HCC from liver inflammatory diseases chronic hepatitis B and C. Importantly, distinct DNA methylation differences emerge at early stages and these might serve as noninvasive diagnostic markers of early stage HCC. The broad differences in DNA methylation that progress with HCC suggest a possible role for epigenetic modulation of the peripheral immune system in HCC and its progression that warrants further exploration.

## Methods

### Patient samples

HCC staging was diagnosed according to EASL–EORTC Clinical Practice Guidelines: Management of hepatocellular carcinoma. The patients were divided into four groups, including stage 0 (1), stage A (2), stage B (3), and stage C+D (4). For simplicity, we refer to stages 1–4 in the figures and manuscript. Chronic hepatitis B diagnosing was confirmed using AASLD practice guideline for chronic hepatitis B, and chronic hepatitis C diagnosing was according to AASLD recommendations for testing, managing, and treating Hepatitis C. A strict exclusion criterion was cirrhosis, any other known inflammatory disease (bacterial or viral infection with the exception of hepatitis B or C, diabetes, asthma, autoimmune disease, active thyroid disease) which could alter T cells and monocyte characteristics as well as presence of other cancers. Clinical characteristics of patients are provided in Tables 1 and 2 and additional information is found in Additional file 14: Table S13, Additional file 15: Table S14 and Additional file 17: Table S16. The participants in the study provided consent according to the regulations of the Capital Medical School. All methods were performed in accordance with the relevant guidelines and regulations. All the candidates were enrolled in the study since 2014 and all the patients prior to receiving the standard therapy according to the BCLC criteria. Whole-blood specimens were collected before the start of standard therapy for the second and third cohort. For the first cohort, blood was drawn either prior to initiation of therapy, prior to surgery, or on the day of surgery (see Additional file 17: Table S16 for details). Informed consent has been obtained from all participants and the study received ethical approval from The Capital Medical School in Beijing and McGill University (IRB Study Number A02-M34-13B).

### Illumina Beadchip 450K analysis

DNA was extracted from T cells isolated using antiCD3 immuno-magnetic beads (Dynabeads Invitrogen), bisulfite converted, and subjected to Illumina HumanMethyaltion450k BeadChip analysis. Samples were randomized with respect to slide and position on arrays, and all samples were hybridized and scanned concurrently to mitigate batch effects as recommended by McGill genome center using Illumina Infinum HD technology user guide. Illumina arrays were analyzed using the ChAMP Bioconductor package in R [46]. IDAT files were used as input in the champ.load function using minfi quality control and normalization options. Raw data were filtered for probes with a detection value of *P* > 0.01 in at least one sample. We filtered out probes on the X or Y chromosome to mitigate sex effects and probes with SNPs as identified in [57], as well as probes that align to multiple locations as identified in [57]. Batch effects were analyzed on the non-normalized data using the function champ.svd. Five out of the first six principal components were associated with group and batch (slides). Intra-array normalization to adjust the data for bias introduced by the Infinium type 2 probe design was performed using beta-mixture quantile normalization (BMIQ) with function champ.norm (norm = “BMIQ”) [46]. We corrected for batch effects after BMIQ normalization using champ.runcombat function. Cell count analysis for white blood cells distribution in our samples was performed according to the Houseman algorithm [54] using the function estimateCellCounts and FlowSorted.Blood.450k data as reference. We used the Beta values of the batch corrected normalized data for downstream statistical analyses. To compute linear correlation between HCC stages and quantitative distribution of DNA methylation at the 450K CG sites, we performed Pearson correlation between the normalized DNA methylation values and stages of HCC (with stage codes of 0 for control, 1 and 2 for hepatitis B and C, respectively, and 3–6 for the four stages of HCC) using the Pearson correlation function in R and correcting for multiple testing using the method “fdr” of Benjamini Hochberg (adjusted *P* value (*Q*) of <0.05) as well as the conservative Bonferroni correction (Q < 1 × 10^−7^). Differentially methylated CGs (MVP) were called using the Bioconductor package Limma [45] as implemented in ChAMP using either “fdr” for multiple testing correction (adjusted *P* value (*Q*) of < 0.05) or Bonferroni corrections. Multifactorial ANOVA with group, sex, and age as cofactors was performed for CGs that were shortlisted for association with HCC using loop_anova lmFit function with Bonferoni adjustment for multiple testing. Multivariate linear regression was performed on the shortlisted CG sites that were found to associate with HCC to test whether these associations will survive if we used cell counts, sex, age, and alcohol abuse as covariates in the linear regression model using the lmFit function in R. Comparison of differentially methylated (relative to control) gene lists in different groups was performed using Venny (Oliveros JC 2007; http://bioinfogp.cnb.csic.es/tools/venny/index.html). Significance of overlap between two groups was determined using hypergeometric Fisher exact test in R. Hierarchical clustering was performed using one minus Pearson correlation, and heatmaps were generated in the Broad institute GeneE application (https://software.broadinstitute.org/GENE-E/).

### Pyrosequencing

Pyrosequencing was performed using the Pyro Mark Q24 (Qiagen) machine, and results were analyzed with Pyro Mark Q24 Software 2.0 (Qiagen). All data were expressed as mean ± standard error of the mean (SEM). The statistical analysis was undertaken using Prism (GraphPad Software Inc., San Diego, California). Primers used for the analysis are listed in Additional file 18: Table S17. All data were analyzed using Student’s *t* test. Significance was set at *P* < 0.05 for comparisons of two groups. When multiple groups were involved, ANOVA followed by Bonferroni corrections for multiple testing were used. We determined using multivariate linear regressions whether confounding clinical variables age, sex, smoking, drinking, or treatment were potential covariates. None of these confounding factors showed consistent correlation with CG methylation across the groups.

## Additional files

Additional file 1: Table S1. CG sites whose quantitative level of DNA methylation correlates with the stage of HCC as determined by a Pearson correlation analysis (*P*<1x10-^7^). (CSV 675 kb)

Additional file 2: Table S2. Differentially methylated sites between Stage 1 HCC and healthy controls. (CSV 929 kb)

Additional file 3: Table S3. Differentially methylated sites between Stage 2 HCC and healthy controls. (CSV 1433 kb)

Additional file 4: Table S4. Differentially methylated sites between Stage 3 HCC and healthy controls. (CSV 2002 kb)

Additional file 5: Table S5. Differentially methylated sites between Stage 4 HCC and healthy controls. (CSV 3562 kb)

Additional file 6: Table S6. Annotated non-redundant list of 350CGs and 369 CGs that are differentially methylated between stages of HCC and healthy controls. (CSV 41 kb)

Additional file 7: Figure S1. Differentially Methylated CG Sites at different stages of HCC and “cross-validation”. a. Heat map presentation of hierarchical clustering of 69 people by 14 differentially methylated CGs between HCC stage 2 and control. b. Heat map of hierarchical clustering of 69 people by 58 differentially methylated CGs between HCC stage 3 and control. **Figure S2.** Differentially Methylated CG Sites at different stages of HCC in a “training set” and “cross-validation” in a “validation set”. a. Heat map presentation of hierarchical clustering of 35 people by a 369 CG signature that correlate with progression in a “training set” (right panel) classify HCC and controls in a “validation set” (left panel as well). b. Heat map of a randomized list of 350 CGs on all patients and controls. **Figure S3.** Prediction of late stage HCC using a penalized model using the 369 CG list which was trained on a randomized half of the HCC patients and controls ("training set") and tested on the other half ("validation set"). The plot shows the “validated” samples (The y axis indicates the predicted probability of late stage HCC for each person (from 0 to 1) (True if prediction >0.5 and False if prediction is <0.5). All late HCC stages in the “validation set” are TRUE and all other stages and controls are FALSE. (PDF 717 kb)

Additional file 8: Table S7. List of CG DNA methylation markers derived from penalized regression model on 350 CG and 369CG sites distinguishing early stages HCC from late stages. (CSV 395 bytes)

Additional file 9: Table S8. Multivariate analysis of 350 CGs. Table provides p values on the right and adjusted values (350 measurements, Bonferroni) on the left. (CSV 95 kb)

Additional file 10: Table S9. Multifactorial ANOVA analysis of 350 CGs. No interaction detected between group (HCC) and sex and age as independent variables with CG methylation as a dependent variable. (CSV 31 kb)

Additional file 11: Table S10. Differentially methylated CG sites in T cell DNA between healthy controls and HCC. (CSV 1586 kb)

Additional file 12: Table S11. Correlation of methylation of CG sites associated with the PD-1 gene and progression of HCC. (CSV 2 kb)

Additional file 13: Table S12. Differentially methylated that correlate with HCC progression in PBMC. (CSV 57 kb)

Additional file 14: Table S13. Clinical data of second cohort. (CSV 11 kb)

Additional file 15: Table S14. Clinical data of third cohort. (XLSX 10 kb)

Additional file 16: Table S15. Descriptive statistics for (Fig. 6a). (XLS 87 kb)

Additional file 17: Table S16. Clinical data of first cohort. (XLSX 18 kb)

Additional file 18: Table S17. Pyrosequencing primers. (CSV 867 bytes)

## Abbreviations

AHNAK: Neuroblast differentiation-associated protein
*AKAP7*: *A Kinase (PRKA) Anchor protein 7*
*CCL1*: Chemokine (C-C motif) ligand 1
*CCL18*: Chemokine (C-C motif) ligand 18
*CCL24*: Chemokine (C-C motif) ligand 24
*CCL7*: Chemokine (C-C motif) ligand 7
*CCR6*: *Chemokine receptor 6*
*CCR7*: *Chemokine receptor 7*
*CCR9*: *Chemokine receptor 9*
*CD14*: *Cluster of differentiation 14*
*CD2*: *Cluster of differentiation 2*
*CD38*: *Cluster of differentiation 38*
*CD44*: *Cluster of differentiation 44*
*CD6*: *Cluster of differentiation 6*
*CD80*: *Cluster of differentiation 80*
*CD83*: *Cluster of differentiation 80*
DMR: Differentially methylated region
EWAS: Epigenome wide association studies
HCC: Hepatocellular carcinoma
HepB: Hepatitis B
HepC: Hepatitis C
*Il10*: *Interleukin 10*
*IL12RB2*: Interleukin 1 receptor B2
*IL16*: *Interleukin 16*
*IL18*: *Interleukin 18*
*Il1B*: *Interleukin 1 beta*
*IL1R1*: *Interleukin1 receptor 1*
*IL1R2*: *Interleukin 1 receptor 2*
*IL2*: Interleukin 2
*Il24*: *Interleukin 24*
*IL2RA*: *Interleukin 2 receptor A*
*IL4*: *Interleukin 4*
*IL4R*: *Interleukin 4 receptor*
*IL5*: *Interleukin5*
*IL5RA*: *Interleukin 5 receptor A*
IL7: Interleukin 7
*NFAT*: *Nuclear factor of activated T-cells*
*NFKB*: *Nuclear factor kappa B*
PBMC: Peripheral blood mononuclear cells
PD-1: Programmed cell death 1
*ROC*: Receiver Operating Characteristic
*SLFN14*: *Schlafen family member 14*
*STAP1*: *Signal Transducing Adaptor Family 1*
*STAT1*: *Signal transducer and activator of transcription 1*
*STAT3*: *Signal transducer and activator of transcription 3*
*TGFbeta3*: *Transforming growth factor beta 3*
*TGFbetaI*: *Transforming growth factor beta 1*
*TNFa*: *Tumor necrosis factor alpha*
*VEGF*: *Vascular endothelial growth factor*
