# Supplementary figures and images for: The signature of liver cancer in immune cells DNA methylation
Source: Clin Epigenetics. 2018 Jan 18;10:8. doi: 10.1186/s13148-017-0436-1 (PMC5774119; doi:10.1186/s13148-017-0436-1)

a

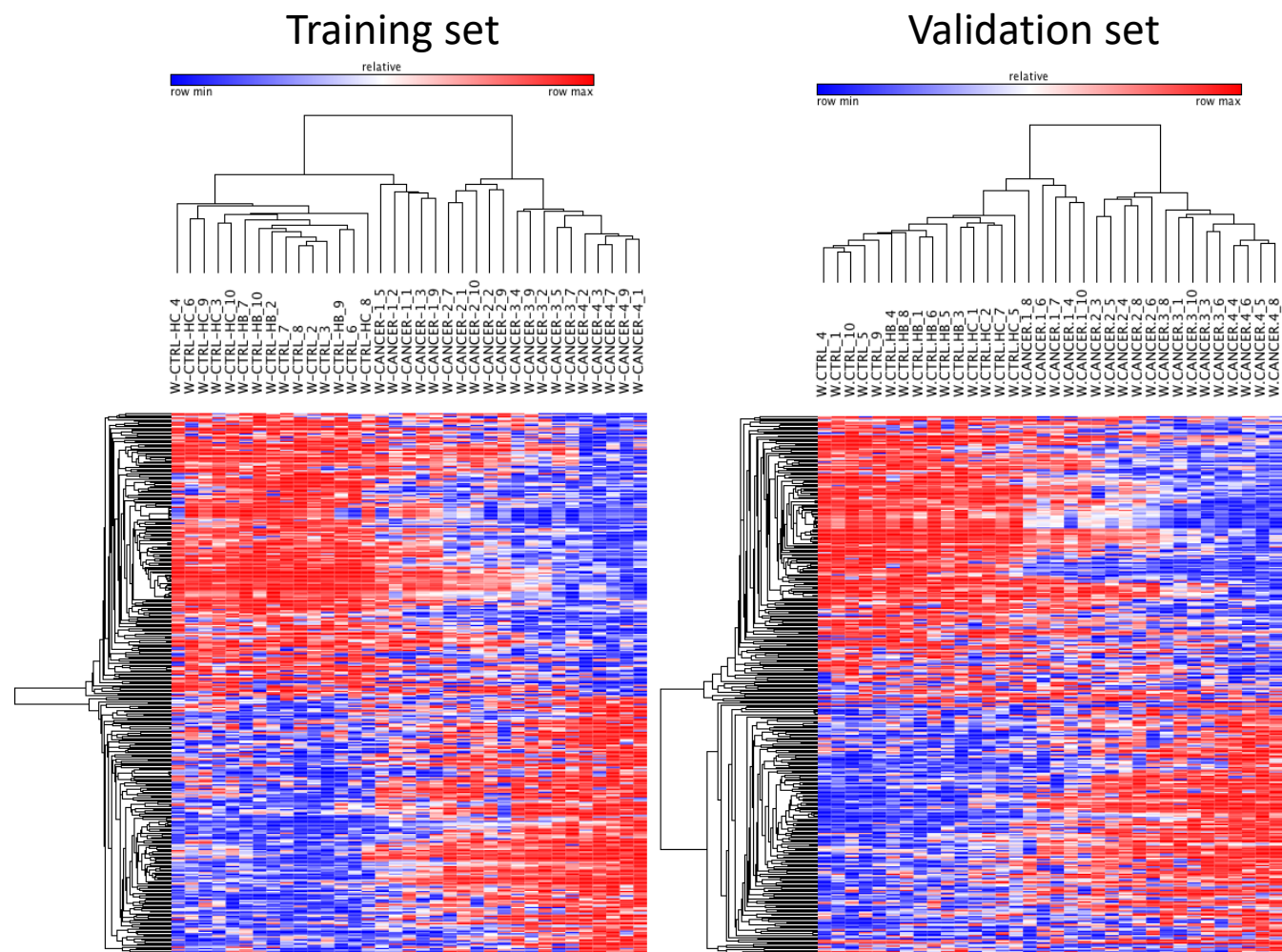

b

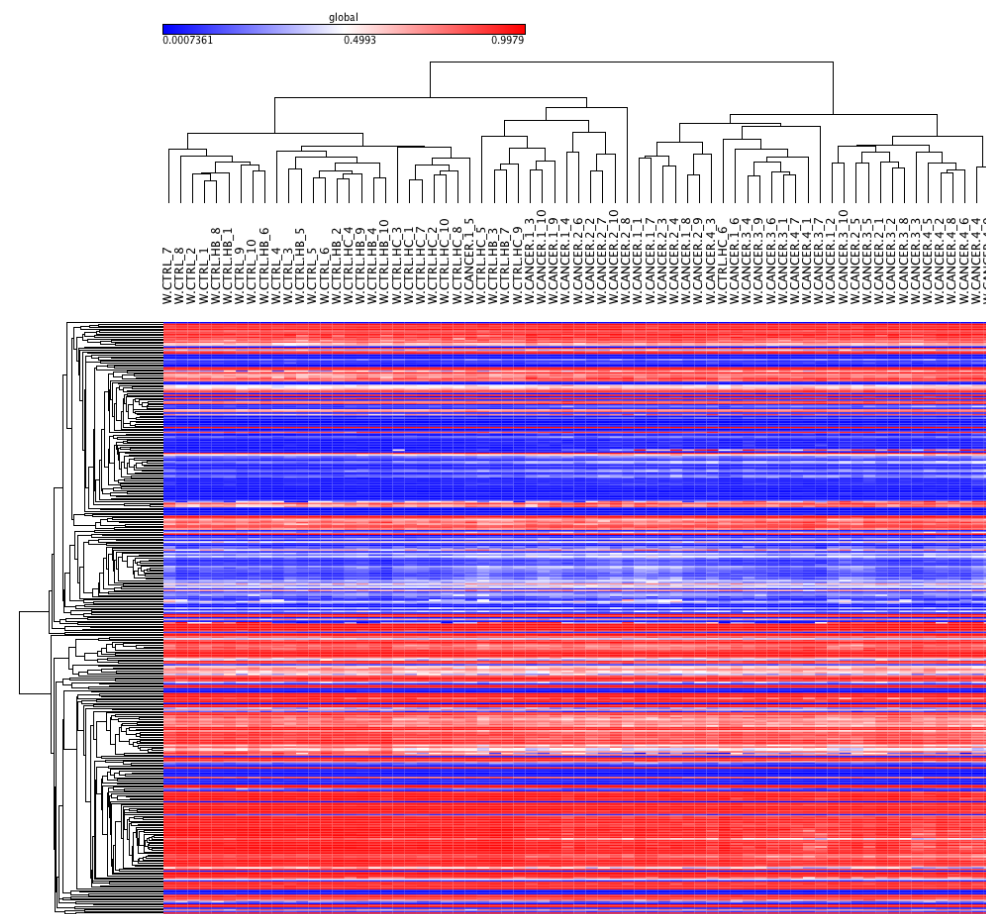

Figure S2

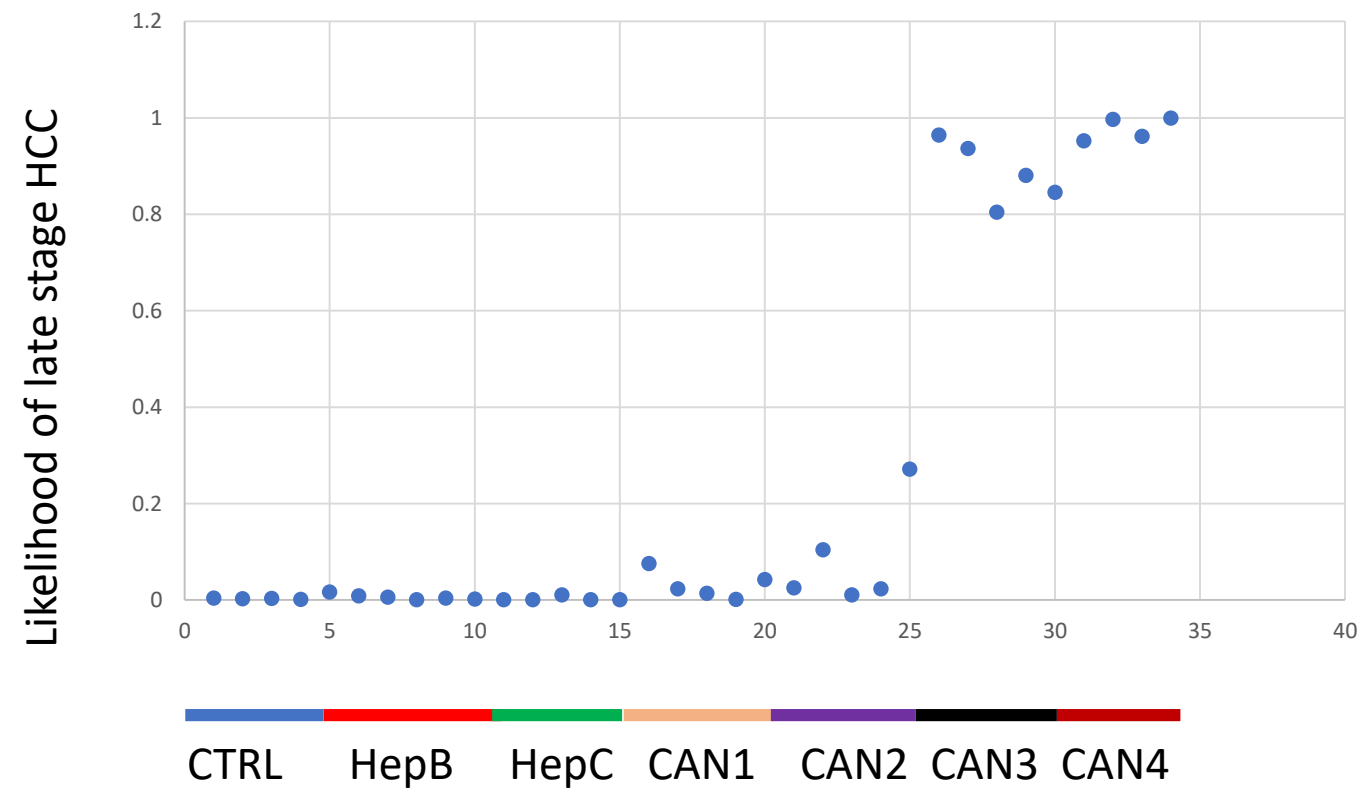

Figure S3

Supplement: Supplementary file 7 — Differentially Methylated CG Sites at different stages of HCC and “cross-validation”. a. Heat map presentation of hierarchical clustering of 69 people by 14 differentially methylated CGs between HCC stage 2 and control. b. Heat map of hierarchical clustering of 69 people by 58 differentially methylated CGs between HCC stage 3 and control. Figure S2. Differentially Methylated CG Sites at different stages of HCC in a “training set” and “cross-validation” in a “validation set”. a. Heat map presentation of hierarchical clustering of 35 people by a 369 CG signature that correlate with progression in a “training set” (right panel) classify HCC and controls in a “validation set” (left panel as well). b. Heat map of a randomized list of 350 CGs on all patients and controls. Figure S3. Prediction of late stage HCC using a penalized model using the 369 CG list which was trained on a randomized half of the HCC patients and controls ("training set") and tested on the other half ("validation set"). The plot shows the “validated” samples (The y axis indicates the predicted probability of late stage HCC for each person (from 0 to 1) (True if prediction >0.5 and False if prediction is <0.5). All late HCC stages in the “validation set” are TRUE and all other stages and controls are FALSE. (PDF 717 kb) [file 13148_2017_436_MOESM7_ESM.pdf]
